# Ethanolic Extract of *Padina arborescens* Suppresses Melanogenesis and Attenuates UVB-Induced Photodamage in Cellular and Zebrafish Models

**DOI:** 10.3390/ijms27083382

**Published:** 2026-04-09

**Authors:** Yun-Su Lee, Wook-Chul Kim, Kyeong Min Lee, Seo-Rin Jung, Seung Tae Im, Min-Cheol Kang, Seung-Hong Lee

**Affiliations:** 1 Department of Medical Science, Soonchunhyang University, Asan 31538, Republic of Korea; yslee9802@naver.com (Y.-S.L.); wookchul0828@naver.com (W.-C.K.); dlrudals8454@naver.com (K.M.L.); jsrpa020329@naver.com (S.-R.J.); 2 Research Group of Food Processing, Korea Food Research Institute, Wanju 55365, Republic of Korea; lama1010@kfri.re.kr; 3 Department of Pharmaceutical Engineering, Soonchunhyang University, Asan 31538, Republic of Korea

**Keywords:** ultraviolet (UV), *Padina arborescens*, HaCaT keratinocytes, B16F10 cells, cosmeceutical

## Abstract

Ultraviolet (UV) irradiation induces complex skin damage, including hyperpigmentation, oxidative stress, and alterations in proteins related to keratinocyte differentiation and epidermal barrier-associated status. This study investigated the multifunctional protective effects of *Padina arborescens* ethanolic extract (PAEE) against skin damage in melanocytes, keratinocytes, and zebrafish. In alpha-melanocyte-stimulating hormone (α-MSH)-stimulated B16F10 cells, PAEE effectively suppressed the protein kinase A (PKA)/cyclic adenosine monophosphate (cAMP) response element-binding protein (CREB) signaling pathway, which was associated with reduced expression of microphthalmia-associated transcription factor (MITF) and tyrosinase, leading to decreased melanin synthesis. PAEE also exhibited photoprotective properties by reducing reactive oxygen species (ROS), inhibiting interleukin-1 beta (IL-1β), and attenuating matrix metalloproteinase-1 (MMP-1) upregulation associated with UVB (ultraviolet B)-induced photodamage in HaCaT keratinocytes. Notably, PAEE restored the UVB-reduced expression of filaggrin and involucrin, representative markers of keratinocyte differentiation and epidermal barrier-associated status, in HaCaT keratinocytes. In zebrafish embryos, PAEE suppressed α-MSH-induced melanin accumulation and UVB-induced ROS generation at non-toxic concentrations. Taken together, these results suggest that PAEE exerts anti-melanogenic and photoprotective effects in cellular and zebrasfish models and may serve as a promising marine-derived ingredient for cosmeceutical applications targeting UVB-related skin damage.

## 1. Introduction

The skin is continuously exposed to ultraviolet (UV) radiation, which is one of the major environmental factors contributing to skin damage [1,2]. Among UV wavelengths, ultraviolet B (UVB, 280–320 nm) primarily affects the epidermis and can induce keratinocyte damage accompanied by changes in barrier-associated markers [3]. UVB also stimulates melanogenic signaling, partly by activating α-melanocyte-stimulating hormone (α-MSH) and the melanocortin 1 receptor (MC1R) [4,5]. MC1R signaling increases cyclic adenosine monophosphate (cAMP) and activates protein kinase A (PKA), which phosphorylates cAMP response element-binding protein (CREB) and induces microphthalmia-associated transcription factor (MITF) expression [6,7]. MITF upregulates melanogenic genes, including tyrosinase and tyrosinase-related protein 1 (TRP-1), thereby promoting melanin synthesis [6,7]. Because MITF is a master regulator of melanogenic gene expression and tyrosinase is the rate-limiting enzyme in melanin synthesis, inhibition of the MITF/tyrosinase axis is widely regarded as a key strategy for controlling hyperpigmentation and developing skin-whitening agents [6,7,8]. In parallel, UVB exposure increases the production of reactive oxygen species (ROS), resulting in oxidative stress and the activation of inflammatory cytokines and matrix metalloproteinases (MMPs) through oxidative stress-responsive signaling pathways, including NF-κB, AP-1, and Nrf2/HO-1-related pathways [9,10,11,12,13]. In particular, IL-1β is widely recognized as a representative pro-inflammatory cytokine involved in UVB-induced skin inflammation [14]. MMP-1 contributes to extracellular matrix degradation and is associated with photodamage [15]. Epidermal barrier homeostasis is supported by multiple structural and junctional proteins, including filaggrin, involucrin, loricrin, and tight-junction-associated proteins [16,17]. Among these, filaggrin and involucrin are widely used as representative markers of keratinocyte differentiation and epidermal barrier-associated status in keratinocyte-based studies [18,19]. UVB irradiation downregulates these proteins, thereby compromising barrier integrity and reducing the skin’s ability to retain moisture [20]. Such UVB-induced changes may contribute to impaired epidermal homeostasis, increased skin sensitivity, and inflammatory responses [21]. Considering the complex mechanisms underlying UVB-induced skin damage, there is a growing demand for multifunctional agents that inhibit melanogenesis while alleviating oxidative and inflammatory stress and ameliorating UVB-responsive epidermal changes [22,23,24].

The increased consumer demand for safe and sustainable products has sparked interest in naturally derived, skin-protective ingredients [25]. Natural materials are generally considered to be low in toxicity and to cause minimal skin irritation. They also exhibit biocompatible biological activities, making them suitable for functional cosmeceuticals intended for long-term use [26,27]. In particular, marine resources have attracted increasing attention as a largely unexplored source of bioactive compounds with potential applications in functional foods, pharmaceuticals, and cosmeceuticals [28,29].

Seaweeds inhabit intertidal zones at the interface between marine and terrestrial environments and are continually exposed to UV radiation, high salinity, and mechanical stress [30,31]. These environmental stressors can promote the production of protective metabolites, including antioxidant-related phlorotannins, as shown in experimental studies of brown algae [32,33]. Metabolites produced through the unique metabolic pathways of seaweed generally have low toxicity and high biocompatibility, making them highly promising as cosmeceutical ingredients [34,35].

Among these seaweeds, *Padina arborescens* (*P. arborescens*) is a brown macroalgal species that inhabits intertidal environments and is repeatedly exposed to intense UV radiation and strong water currents, environmental conditions that may favor the production of diverse bioactive compounds [36,37]. *P. arborescens* has long been used in traditional medicine, and recent studies have reported various biological activities, including antioxidant, glucose-regulatory, and lipid-lowering effects [38,39,40]. Taken together, these characteristics suggest that *P. arborescens* may have potential as a marine-derived resource for skin photoprotection. However, although previous studies have suggested the biological potential of Padina species and other marine algal extracts in antioxidant, anti-melanogenic, and photoprotective applications, the role of PAEE in UVB-induced skin damage remains insufficiently characterized. In particular, limited evidence is available integrating melanogenesis-related responses, oxidative stress, inflammatory mediators, matrix-degrading factors, and markers of keratinocyte differentiation and epidermal barrier-associated status within a single experimental framework. Moreover, the effects of PAEE on both pigmentation-related and UVB-induced photodamage-related responses have not been comparatively evaluated using complementary in vitro and zebrafish models. Therefore, the present study was designed to investigate the anti-melanogenic and photoprotective effects of PAEE in α-MSH-stimulated B16F10 cells, UVB-irradiated HaCaT keratinocytes, and zebrafish models, with particular focus on melanin accumulation, oxidative stress, inflammation, MMP-1 expression, and markers of keratinocyte differentiation and epidermal barrier-associated status.

## 2. Results

### 2.1. LC-Q-TOF-MS/MS Analysis of Ethanolic Extract from P. arborescens (PAEE)

**Figure 1:**
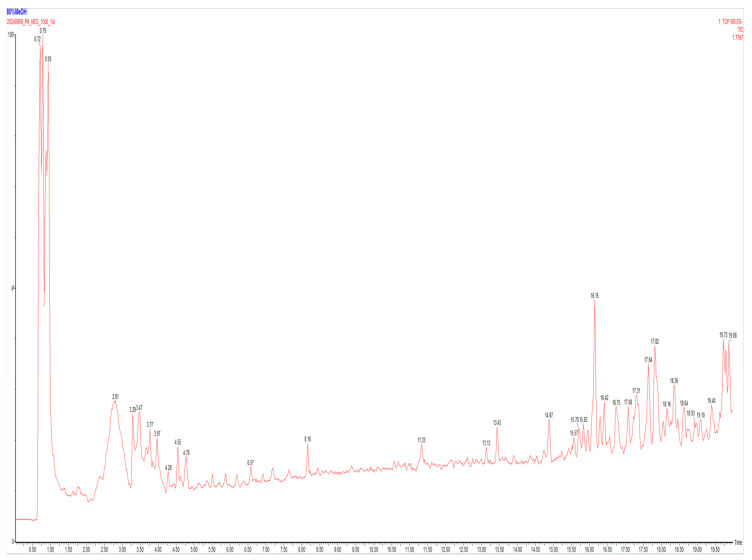
LC-Q-TOF-MS/MS analysis of PAEE. The red trace represents the total ion chromatogram (TIC), and the numbers above the peaks indicate the retention times (min).

Brown algae are a valuable source of bioactive metabolites, including polyphenols and lipid-derived compounds [37]. To characterize the major chemical features present in PAEE, LC-Q-TOF-MS/MS profiling was performed, and 15 representative features were tentatively identified based on detection intensity, mass accuracy, and library-match scores. The LC-MS/MS chromatogram and compound profiles of PAEE are shown in Figure 1 and Table 1. LC-Q-TOF-MS/MS profiling tentatively identified representative metabolites in PAEE, including 2-O-(α-D-glucopyranosyl)-D-glyceric acid, 10,16-dihydroxyhexadecanoic acid, and 1,6-dihydroxycyclohexa-2,4-diene-1-carboxylic acid (Table 1). Overall, the profile suggested the presence of glycosylated acids, hydroxy fatty acid derivatives, and phenolic-related compounds’ structural classes that have been associated with antioxidant activity and may contribute to skin-related bioactivities [41].

Taken together, these findings indicate that PAEE contains structurally diverse metabolites, which may contribute to its observed biological activities.

### 2.2. Cytotoxicity of PAEE in B16F10 Cells and HaCaT Cells

**Figure 2:**
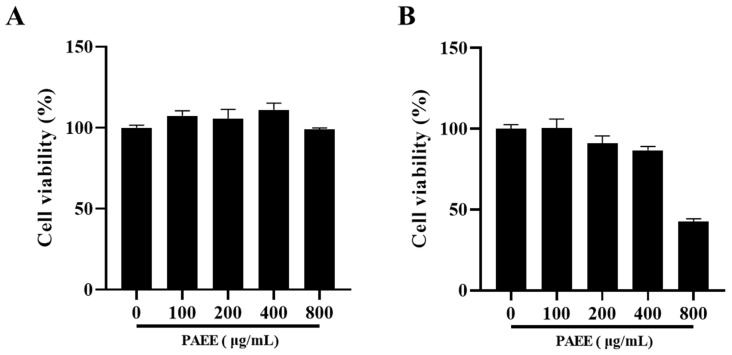
Cytotoxicity of PAEE in B16F10 cells (**A**) and HaCaT cells (**B**). These values are expressed as the mean ± SD of triplicate experiments.

Cell viability was assessed after exposing B16F10 cells and HaCaT cells to various concentrations of PAEE. PAEE showed no significant cytotoxicity in B16F10 cells at concentrations up to 800 μg/mL or in HaCaT cells at concentrations up to 400 μg/mL. B16F10 cells showed no apparent cytotoxicity within the tested concentration range, whereas HaCaT viability decreased to ≤50% at 800 μg/mL (Figure 2). Accordingly, concentrations that did not significantly reduce cell viability were used for subsequent experiments (B16F10: ≤800 μg/mL; HaCaT: ≤400 μg/mL).

### 2.3. Inhibitory Effects of PAEE on Melanogenesis and Tyrosinase Activity Through the Downregulation of Melanin-Producing Pathways in α-MSH-Stimulated B16F10 Cells

**Figure 3:**
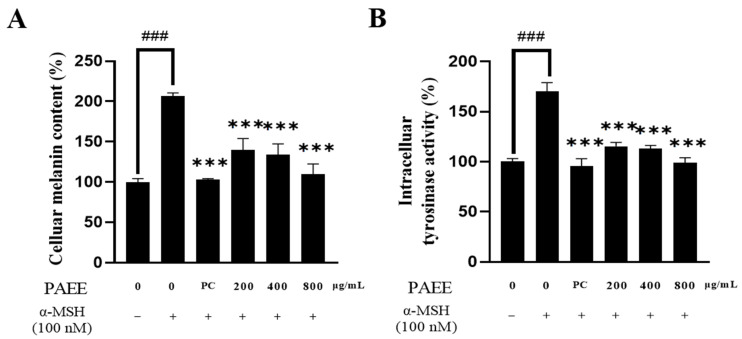
Effect of PAEE on cellular melanin content (**A**) and cellular tyrosinase activity (**B**) in B16F10 melanoma cells. PC denotes the positive control, arbutin (200 μg/mL). Minus (−) and plus (+) signs indicate the absence and presence of the indicated treatment, respectively. Data are expressed as the mean ± SD of triplicate experiments; ### indicates significant differences compared with the control group (*p* < 0.001), and *** indicates significant differences compared with the α-MSH-treated group (*p* < 0.001), as determined by one-way ANOVA followed by Dunnett’s multiple comparisons test.

**Figure 4:**
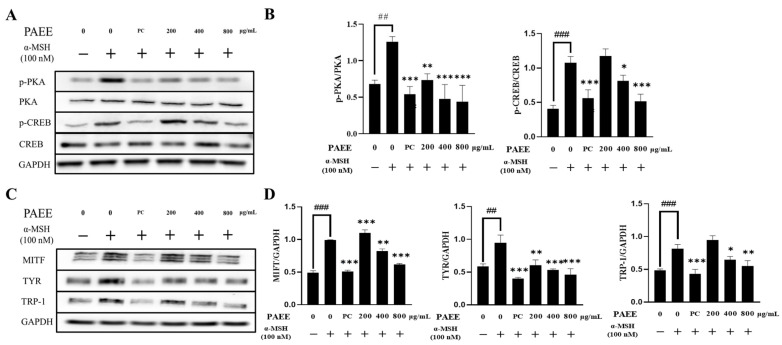
Representative Western blot analysis of total protein expression of CREB, p-CREB, PKA, and p-PKA in B16F10 cells (**A**) and TRP-1, TYR, MITF, and GAPDH in B16F10 cells (**C**). Quantification of protein expression of p-CREB/CREB and p-PKA/PKA in B16F10 cells (**B**) and TRP-1/GAPDH, TYR/GAPDH, and MITF/GAPDH in B16F10 cells (**D**). PC denotes the positive control, arbutin (200 μg/mL). Minus (−) and plus (+) signs indicate the absence and presence of the indicated treatment, respectively. The values are expressed as mean ± SD of triplicate experiments; ##, ### indicate significant differences compared with the control group (*p* < 0.01 and *p* < 0.001, respectively), and *, **, *** indicate significant differences compared with the α-MSH-treated group (*p* < 0.05, *p* < 0.01, and *p* < 0.001, respectively), as determined by one-way ANOVA followed by Dunnett’s multiple comparisons test.

α-MSH promotes melanogenesis via the cAMP/PKA-CREB-MITF axis, which transcriptionally upregulates tyrosinase and tyrosinase-related proteins such as TRP-1 [42]. Therefore, we evaluated whether PAEE modulates this pathway in B16F10 cells stimulated with α-MSH. α-MSH markedly increased cellular melanin content and tyrosinase activity, whereas PAEE significantly reduced both endpoints in a concentration-dependent manner (Figure 3). At lower concentrations, the inhibitory effect was weaker than that of the positive control (arbutin). However, the highest PAEE concentration produced a comparable effect to arbutin. In addition, α-MSH increased the phosphorylation of PKA and CREB, as well as upregulating the expression of MITF, tyrosinase, and TRP-1 proteins. In contrast, PAEE treatment reversed these changes in a dose-dependent manner (Figure 4). These results suggest that PAEE exerts anti-melanogenic effects associated with attenuation of melanogenesis-related signaling and reduced tyrosinase activity. Arbutin was used as a reference anti-melanogenic control to benchmark functional inhibition, rather than to indicate a mechanism identical to that of PAEE.

### 2.4. Effect of PAEE on ROS Production and MMP-1 and IL-1β Expression in UVB-Irradiated HaCaT Cells

**Figure 5:**
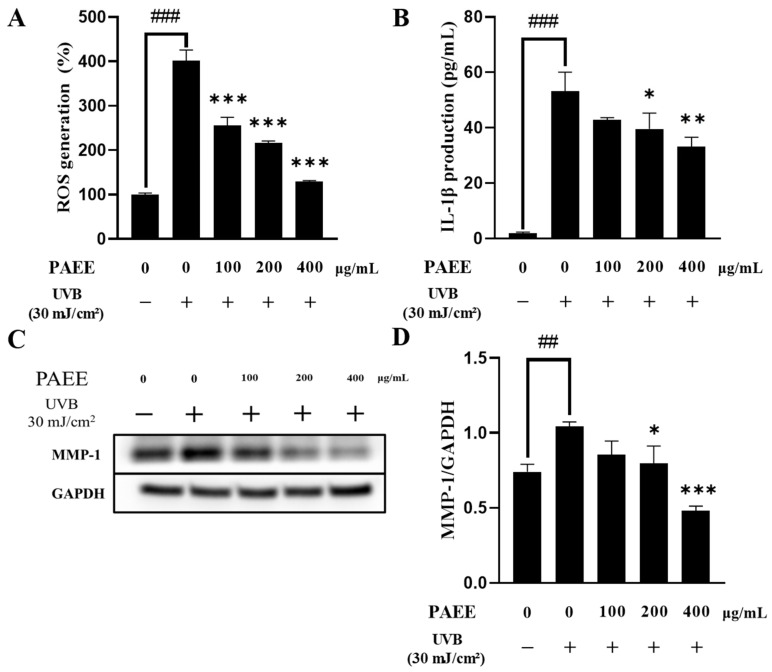
Effect of PAEE on ROS production (**A**) and IL-1β levels (**B**) in HaCaT cells following UVB irradiation. Representative image of Western blot analysis of total protein expression of MMP-1 and GAPDH (**C**) and quantification of protein expression of MMP-1/GAPDH in HaCaT cells (**D**). Cells were treated with 30 mJ/cm^2^ of UVB, followed by PAEE treatment. ROS levels were measured using the DCFH-DA assay, and IL-1β levels were measured by ELISA. Minus (−) and plus (+) signs indicate the absence and presence of the indicated treatment, respectively. The values are expressed as mean ± SD of triplicate experiments; ##, ### indicate significant differences compared with the control group (*p* < 0.01 and *p* < 0.001, respectively), and *, **, *** indicate significant differences compared with the UVB-treated group (*p* < 0.05, *p* < 0.01, and *p* < 0.001, respectively), as determined by one-way ANOVA followed by Dunnett’s multiple comparisons test.

UVB radiation induces cellular stress and accelerates skin aging by increasing intracellular ROS [38]. In HaCaT cells, exposure to UVB markedly increased intracellular ROS levels, whereas PAEE treatment reduced ROS levels in a dose-dependent manner (Figure 5A). ROS can activate various inflammatory signaling pathways, thereby stimulating the production of pro-inflammatory cytokines such as IL-1β. This inflammatory response is also associated with the upregulation of MMP-1, which contributes to tissue damage and photodamage-related extracellular matrix remodeling [39]. UVB irradiation markedly increased IL-1β levels in HaCaT cells, whereas PAEE treatment significantly reduced IL-1β levels in a concentration-dependent manner (Figure 5B). UVB irradiation also increased MMP-1 expression, and PAEE at 400 μg/mL markedly attenuated this induction (Figure 5C). Taken together, these results suggest that PAEE mitigates UVB-induced photodamage by suppressing the generation of ROS, reducing levels of pro-inflammatory cytokine IL-1β, and attenuating MMP-1 upregulation.

### 2.5. Effect of PAEE on Filaggrin and Involucrin Expression in UVB-Irradiated HaCaT Cells

**Figure 6:**
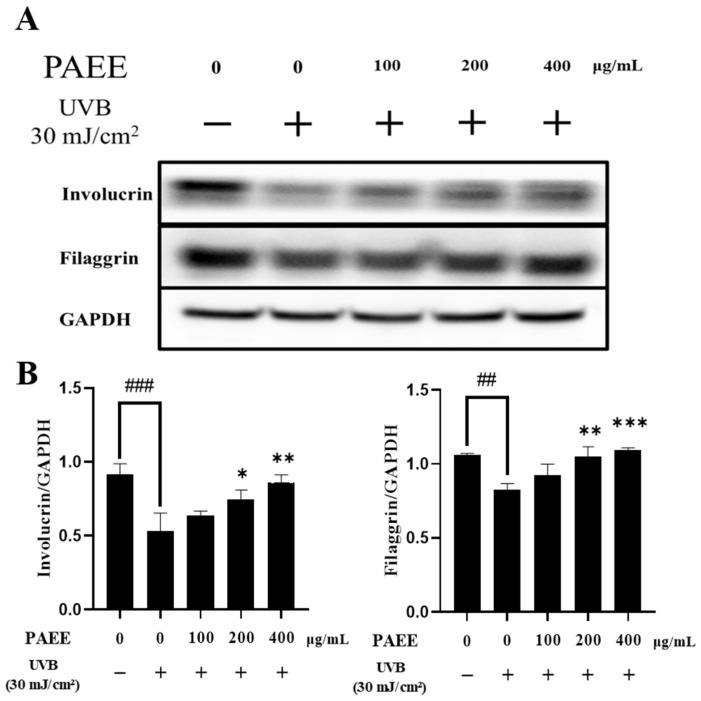
Representative Western blot analysis of total protein expression of involucrin, filaggrin, and GAPDH in HaCaT cells: (**A**) Quantification of protein expression of involucrin/GAPDH and filaggrin/GAPDH (**B**). Minus (−) and plus (+) signs indicate the absence and presence of the indicated treatment, respectively. The values are expressed as mean ± SD of triplicate experiments; ##, ### indicate significant differences compared with the control group (*p* < 0.01 and *p* < 0.001, respectively), and *, **, *** indicate significant differences compared with the UVB-treated group (*p* < 0.05, *p* < 0.01, and *p* < 0.001, respectively), as determined by one-way ANOVA followed by Dunnett’s multiple comparisons test.

UVB-induced oxidative stress and related inflammation can downregulate structural proteins such as filaggrin and involucrin, which are commonly used as representative markers of keratinocyte differentiation and epidermal barrier-associated status in keratinocyte studies [43,44]. To evaluate whether PAEE mitigates UVB-induced reductions in these marker proteins, we assessed the levels of filaggrin and involucrin by Western blotting in UVB-irradiated HaCaT cells. UVB irradiation (30 mJ/cm^2^) markedly decreased filaggrin and involucrin expression levels. However, treatment with PAEE significantly restored both proteins in a concentration-dependent manner, with the strongest effect observed at 400 μg/mL (Figure 6). These findings indicate that PAEE restored the expression of filaggrin and involucrin, representative markers of keratinocyte differentiation and epidermal barrier-associated status, in UVB-irradiated HaCaT cells.

### 2.6. Effects of PAEE on Toxicity in Zebrafish Embryos

**Figure 7:**
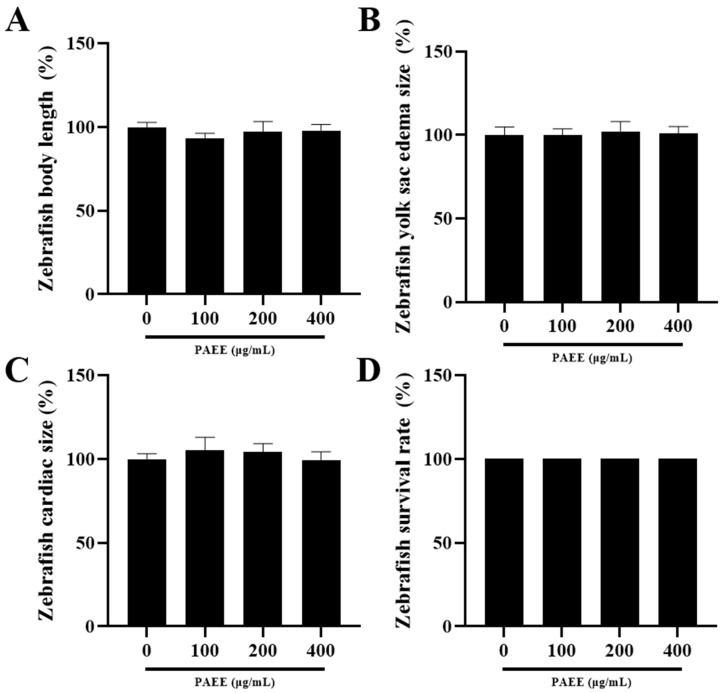
Quantification of morphological toxicity (**A**–**C**) and survival rate (**D**) of zebrafish embryos treated with PAEE. Morphological toxicity was evaluated by body length (**A**), yolk sac edema size (**B**), and cardiac size (**C**), and the survival rate is shown in (**D**). Each condition used n = 5 embryos. Morphological parameters were quantified using ImageJ (version 1.53e). Morphological toxicity values are expressed as mean ± SD (n = 5). Survival rate is presented as the percentage of surviving embryos (n = 5).

To establish a safe concentration range for in vivo experiments, we evaluated PAEE toxicity in zebrafish embryos using survival and morphological endpoints (body length, yolk edema, and cardiac size) [45]. PAEE at 100–400 μg/mL maintained survival rates above 90% and did not induce significant morphological abnormalities compared with controls (Figure 7). In contrast, 800 μg/mL reduced survival to below 50%, indicating toxicity at this concentration. Therefore, 100, 200, and 400 μg/mL were used in subsequent zebrafish assays.

### 2.7. Effects of PAEE on Melanin Accumulation in α-MSH-Induced Zebrafish Embryos

**Figure 8:**
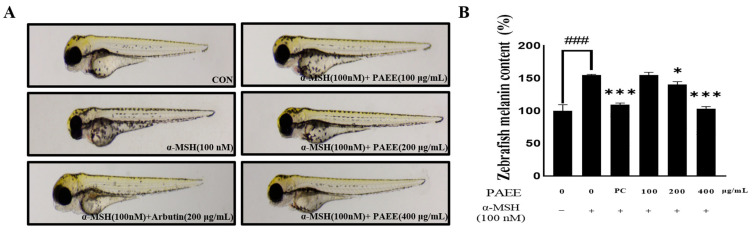
The melanogenic inhibition effect of melanin synthesis by PAEE in zebrafish. Representative image (**A**) and quantification (**B**). PC denotes the positive control, arbutin (200 μg/mL). Minus (−) and plus (+) signs indicate the absence and presence of the indicated treatment, respectively. The values are expressed as mean ± SD of triplicate experiments. ### indicates significant differences compared with the control group (*p* < 0.001), and *, *** indicate significant differences compared with the α-MSH-treated group (*p* < 0.05, *p* < 0.001, respectively), as determined by one-way ANOVA followed by Dunnett’s multiple comparisons test.

Zebrafish larvae were treated with α-MSH to induce pigmentation, and the ability of PAEE to suppress melanin deposition was evaluated. PAEE dose-dependently reduced α-MSH-induced melanin accumulation (Figure 8). Quantitative analysis confirmed that PAEE significantly decreased melanin content relative to the α-MSH group, with the strongest inhibition at 400 μg/mL. At this concentration, the effect was comparable to or slightly greater than that of the positive control (arbutin). These data indicate that PAEE exerts in vivo anti-melanogenic activity in zebrafish.

### 2.8. Effect of PAEE on ROS Levels in UVB-Irradiated Zebrafish Embryos

**Figure 9:**
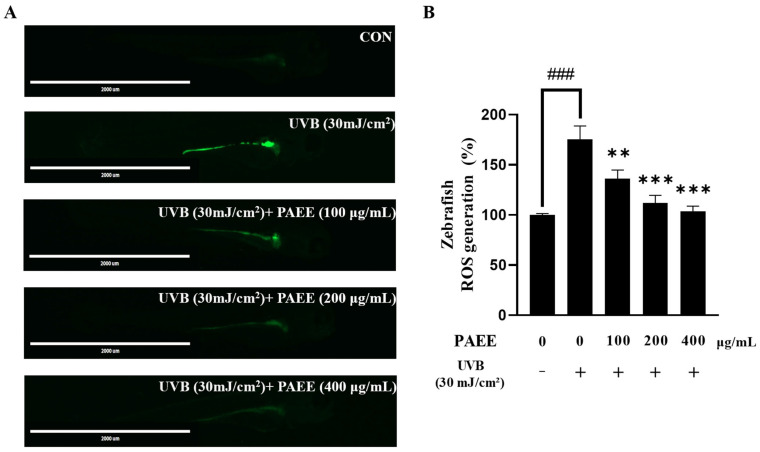
Analysis of ROS production in zebrafish larvae following UVB exposure and PAEE treatment using the DCFH-DA fluorescence assay. Representative image: (**A**) Quantification of DCFH-DA fluorescence intensity (**B**). Minus (−) and plus (+) signs indicate the absence and presence of the indicated treatment, respectively. The values are expressed as mean ± SD of triplicate experiments; ### indicates significant differences compared with the control group (*p* < 0.001), and **, *** indicate significant differences compared with the 30 mJ/cm^2^ UVB-treated group (*p* < 0.01 and *p* < 0.001, respectively), as determined by one-way ANOVA followed by Dunnett’s multiple comparisons test.

UVB exposure generates excessive ROS in developing zebrafish embryos [46]. Accordingly, ROS levels were assessed using the DCFH-DA assay to determine whether PAEE alleviates UVB-induced oxidative damage in an in vivo zebrafish model. UVB irradiation markedly increased ROS-associated fluorescence, indicating oxidative stress, whereas PAEE reduced fluorescence intensity in a dose-dependent manner (Figure 9). These results suggest that PAEE alleviates UVB-induced oxidative stress in vivo.

## 3. Discussion

Taken together, the present study showed that PAEE reduced melanogenesis-related responses in α-MSH-stimulated B16F10 cells, attenuated UVB-induced oxidative and inflammatory responses in HaCaT cells, restored the expression of representative markers of keratinocyte differentiation and epidermal barrier-associated status, and showed complementary anti-melanogenic and antioxidant effects in zebrafish.

The skin functions as a barrier against external stimuli and is continuously exposed to UV radiation, a major driver of photodamage, hyperpigmentation, oxidative stress, inflammation, and barrier-associated changes [47,48,49,50,51]. In this context, multifunctional ingredients capable of modulating several UVB-related responses are of considerable interest [24,25]. Because marine brown algae are repeatedly exposed to environmental stress, which can promote the biosynthesis or accumulation of protective metabolites, *P. arborescens* may represent a promising source of structurally diverse bioactive compounds with skin-related bioactivity [34,35,36,37,38,39]. Here, we evaluated the cosmeceutical potential of *P. arborescens* ethanolic extract (PAEE) using α-MSH- and UVB-induced cellular models, along with zebrafish assays of pigmentation and oxidative stress.

LC-Q-TOF-MS/MS profiling suggested that PAEE contains low-molecular-weight compounds with phenolic moieties, glycosidic linkages, and hydroxy fatty acid chains. Because these structural classes are frequently associated with antioxidant and anti-inflammatory properties, the chemical profile provides plausible support for the observed bioactivities of PAEE [52,53]. However, because the annotations were tentative and not confirmed by authentic standards or NMR, the present LC-Q-TOF-MS/MS data should be interpreted as exploratory compositional profiling rather than definitive compound identification. The hormone α-MSH secreted by the skin following UV irradiation is a well-established inducer of melanogenesis. Upon binding to MC1R, α-MSH elevates intracellular cAMP and activates the PKA–CREB–MITF axis, thereby upregulating tyrosinase and TRP-1 and promoting melanin synthesis [48].

In the present study, α-MSH treatment increased the phosphorylation of PKA and CREB in B16F10 cells, as well as upregulating the protein levels of MITF and TRP-1. However, PAEE attenuated these α-MSH-induced melanogenesis-related factors in a dose-dependent manner. These results suggest that PAEE downregulates melanogenesis by decreasing tyrosinase activity and modulating upstream signaling to affect melanogenic factor expression. Consistently, both intracellular melanin content and tyrosinase activity were downregulated, demonstrating concordance between the proposed mechanism and the observed functional results. Although the overall pattern was consistent with attenuation of melanogenesis-related signaling, not all signaling intermediates were uniformly affected at the intermediate concentration, and MITF did not show a fully monotonic decrease across all doses. This may reflect a non-linear response pattern of a complex crude extract rather than a simple single-compound dose–response relationship. Notably, PAEE showed potent inhibitory effects at the highest tested concentration, demonstrating an inhibitory effect comparable to that of the positive control, arbutin. These findings suggest that PAEE may be a valuable marine-derived source of anti-melanogenic activity for further cosmeceutical investigation.

Ultraviolet (UV) irradiation induces excessive generation of ROS in the skin, leading to oxidative stress that disrupts epidermal barrier integrity [11]. Consequently, excessive ROS can induce pro-inflammatory cytokines such as IL-1β, disrupt water retention, and downregulate filaggrin and involucrin expression, ultimately causing dryness and inflammation [54,55]. ROS can also increase MMP-1 expression, which degrades collagen and contributes to extracellular matrix damage and photoaging [56,57]. The present study found that PAEE significantly reduced UVB-induced increases in ROS, IL-1β, and MMP-1 in HaCaT cells. Interestingly, PAEE more consistently reduced ROS than IL-1β, suggesting that its antioxidant effect may be more robust than its cytokine-level anti-inflammatory effect under the present conditions. This difference may reflect distinct signaling thresholds or temporal kinetics between oxidative and inflammatory responses [58]. The magnitude of ROS reduction did not exactly parallel the change in MMP-1 expression, which may indicate that MMP-1 regulation is influenced not only by ROS levels at a single time point but also by broader downstream signaling and transcriptional control [59]. These results suggest that PAEE may alleviate UVB-induced photodamage by reducing oxidative and inflammatory responses. Although the precise upstream mechanism was not investigated in this study, the reduction in ROS, IL-1β, and MMP-1 suggests that PAEE may influence ROS-sensitive signaling pathways involved in UVB-induced photodamage, such as Nrf2/HO-1, MAPK/AP-1, and NF-κB-related pathways [12]. However, direct pathway-focused analyses were not performed, and this mechanistic interpretation should therefore be considered preliminary. Furthermore, PAEE alleviated the UVB-induced reduction in filaggrin and involucrin expression. Although PAEE restored filaggrin and involucrin expression, these findings should be interpreted as marker-level evidence related to keratinocyte differentiation and epidermal barrier-associated status rather than direct proof of barrier function recovery, because functional barrier assays were not performed in this study. Considering the association between ROS generation, inflammation, matrix protein degradation, and the loss of filaggrin and involucrin expression, the antioxidant and cytoprotective effects of PAEE may be linked to changes in markers of keratinocyte differentiation and epidermal barrier-associated status. Taken together, the recovery of filaggrin and involucrin expression and the reduction in oxidative stress suggest that PAEE may modulate UVB-responsive epidermal changes under the present experimental conditions.

Zebrafish (*Danio rerio*) are increasingly being used as a practical in vivo model for the simultaneous evaluation of toxicity and functional efficacy, and they have been widely used in the screening of functional ingredients [60]. In addition to cell-based experiments, zebrafish embryos provided complementary in vivo evidence regarding the efficacy and safety of PAEE. In this study, toxicity was assessed by combining overall survival with morphological toxicology indicators, including body length as an indicator of growth retardation, cardiac size as an indicator of cardiotoxicity or edema-related changes, and yolk edema size as an indicator of developmental and fluid balance disturbances [61,62]. In α-MSH-treated zebrafish, PAEE decreased melanin accumulation; in UVB-exposed zebrafish, it decreased ROS fluorescence in a concentration-dependent manner. Furthermore, no overt toxicity was observed under the tested conditions, indicating that PAEE showed beneficial organism-level effects within a relatively safe concentration range under the present experimental conditions. Although zebrafish provide useful organism-level evidence, their skin structure and physiology differ from those of mammalian and human skin. Therefore, these findings should be interpreted as supportive preclinical evidence rather than direct evidence of efficacy in human skin.

### 3.1. Limitations of Study

This study has several limitations. First, the biological effects of PAEE were evaluated in B16F10 and HaCaT cell lines, together with complementary zebrafish assays, which do not fully recapitulate human skin physiology. Second, the chemical characterization of PAEE was preliminary and did not identify the active constituents responsible for the observed effects. Third, ROS measurement relied on DCFH-DA; inflammatory evaluation was limited to IL-1β; and direct assessment of intracellular cAMP, collagen degradation, extracellular matrix remodeling, and functional barrier recovery was not performed. Fourth, only arbutin was used as a reference anti-melanogenic control in the present study. Finally, the zebrafish experiments used a relatively small sample size for morphological endpoints.

### 3.2. Future Directions

Future studies should identify the active constituents of PAEE through bioactivity-guided fractionation, validate the findings in primary human melanocytes and reconstructed human skin models, and further investigate the upstream mechanisms associated with oxidative stress and inflammation. In addition, future work should include broader inflammatory cytokines, such as IL-6 and TNF-α, as well as more specific oxidative stress markers, direct assessment of cAMP signaling, collagen-related outcomes, and functional barrier assays. Further studies should also include larger zebrafish experiments with increased sample sizes and more detailed image-based evaluation of both pigmentation and UVB-induced photodamage-related responses.

### 3.3. Conclusions

This study suggests that an ethanolic extract of the brown alga *P. arborescens* (PAEE) exhibits both anti-melanogenic and photoprotective activities. In B16F10 cells, PAEE inhibited α-MSH-induced melanogenesis by attenuating cAMP/PKA–CREB–MITF-related signaling and downregulating melanogenic enzymes. In HaCaT keratinocytes, PAEE alleviated UVB-induced oxidative stress and inflammatory responses and restored the expression of representative markers of keratinocyte differentiation and epidermal barrier-associated status. In complementary zebrafish assays, PAEE reduced melanin accumulation and ROS levels. Overall, these results suggest that PAEE has potential as a multifunctional marine-derived candidate that modulates melanogenesis- and photodamage-related responses under the present experimental conditions. However, these findings are limited to B16F10 and HaCaT cell lines and zebrafish embryos, and further validation in primary human cells, reconstructed human skin models, and mammalian systems is required.

## 4. Materials and Methods

### 4.1. Chemicals and Reagents

Ethanol was purchased from Duksan Pure Chemicals Co., Ltd. (Ansan, Republic of Korea). MTT [3-(4,5-dimethylthiazol-2-yl)-2,5-diphenyltetrazolium bromide], 2′,7′-dichlorodihydrofluorescein diacetate (DCFH-DA), Triton X-100, and PMSF (phenylmethylsulfonyl fluoride) were purchased from Sigma-Aldrich (St. Louis, MO, USA).

Antibodies against phosphorylated PKA catalytic subunit (Thr197) (p-PKA C, #4781), total PKA C-α (#4782), phosphorylated CREB (Ser133) (p-CREB, #9198), total CREB (#9197), and MITF (D3B4T, #97800) were purchased from Cell Signaling Technology (Danvers, MA, USA). Antibodies against tyrosinase (#sc-20035), TRP-1 (#sc-58438), involucrin (#sc-53361), filaggrin (#sc-66192), and mouse-derived GAPDH (#sc-32233) were purchased from Santa Cruz Biotechnology (Santa Cruz, CA, USA). The antibody against MMP-1 (10371-2-AP) was purchased from Proteintech (Rosemont, IL, USA). Dulbecco’s modified Eagle’s medium (DMEM), penicillin/streptomycin, fetal bovine serum (FBS), phosphate-buffered saline (PBS), and trypsin–EDTA were obtained from Gibco BRL (Thermo Fisher Scientific, Waltham, MA, USA). All other chemicals and reagents used in this study were of analytical grade.

### 4.2. Preparation of Ethanolic Extract from Padina arborescens (PAEE)

*P. arborescens* samples were obtained from the coastal region of Jeju Island, Korea. After removal of salts and debris by washing with tap water, the samples were freeze-dried and pulverized. The powdered material was extracted with 70% ethanol (1:10, *w*/*v*) for 24 h at room temperature under gentle agitation. The extract was filtered through Whatman No. 2 filter paper, concentrated using a rotary evaporator (Eyela N-1000; Tokyo Rikakikai Co., Ltd., Tokyo, Japan), and then freeze-dried to obtain the final extract. The freeze-dried extract was stored at 4 °C and reconstituted immediately before use. The extraction yield of PAEE was 18.0% (*w*/*w*), calculated based on the dry weight of the freeze-dried extract relative to the dry weight of the starting material.

### 4.3. LC-Q-TOF-MS/MS Analysis of Ethanolic Extract from P. arborescens (PAEE)

To profile the chemical constituents of PAEE, 10 mL of 80% methanol (Daejung Chemicals, Cheongju, Republic of Korea) was added to 1 g of PAEE. After centrifugation at 12,000 rpm for 10 min, the resulting supernatant was collected for analysis. The supernatant was analyzed using a liquid chromatography–quadrupole time-of-flight mass spectrometry (LC-Q-TOF MS) system (Waters Corp., Milford, MA, USA). A WATERS BEH C18 column (2.1 mm × 100 mm, 1.7 μm; Waters, Milford, MA, USA) was used for chromatographic separation. The mobile phases consisted of 0.1% formic acid in water (solvent A) and 0.1% formic acid in acetonitrile (solvent B), with a flow rate of 0.3 mL/min at a column temperature of 40 °C. Eluted compounds were analyzed in positive electrospray ionization (ESI) mode using Q-TOF MS. The MS parameters were set as follows: mass range of 100–1300 m/z, capillary voltage of 3 kV, desolvation gas flow of 600 L/h, desolvation temperature of 250 °C, and source temperature of 100 °C. Data processing was carried out using Progenesis QI software (version 2.4; Waters, Milford, MA, USA), and compound identification was performed using the integrated ChemSpider database (Royal Society of Chemistry, Cambridge, UK).

### 4.4. Cell Culture

Human keratinocyte (HaCaT) cells were purchased from the Korean Cell Line Bank (KCLB, Seoul, Republic of Korea), and B16F10 mouse melanoma cells were obtained from the American Type Culture Collection (ATCC; Manassas, VA, USA). Both cell lines were cultured in Dulbecco’s modified Eagle’s medium (DMEM) supplemented with 10% fetal bovine serum (FBS) and 1% antibiotics (penicillin and streptomycin) at 37 °C in a humidified incubator with 5% CO_2_.

### 4.5. UVB Irradiation

HaCaT cells were washed with phosphate-buffered saline (PBS) and irradiated with UVB at a dose of 30 mJ/cm^2^ in the presence of PBS using a UV light meter (UV Lamp, VL-6LM; Vilber, Paris, France) with an emission spectrum of 280–320 nm. The UVB dose (30 mJ/cm^2^) was selected based on previous studies [63] and preliminary optimization because it induced measurable oxidative and barrier-associated responses without causing excessive cytotoxicity under our experimental conditions. Following irradiation, PBS was removed, and the cells were incubated in serum-free DMEM containing the indicated sample under standard culture conditions.

### 4.6. Measurement of Cell Viability

B16F10 cells and HaCaT cells were used to evaluate the cytotoxicity of PAEE using a colorimetric MTT assay [64]. In brief, HaCaT cells were seeded in a 24-well plate at a density of 1.0 × 10^5^ cells/well, and B16F10 cells were seeded in a 24-well plate at a density of 1.0 × 10^4^ cells/well, followed by overnight incubation. After incubation, cells were treated with PAEE (0–800 μg/mL) for 24 h (HaCaT) or 72 h (B16F10). MTT solution was then added to each well and incubated for 3 h at 37 °C. The medium was subsequently removed, and DMSO was added to dissolve the formazan crystals. The plate was incubated overnight, and absorbance was measured at 540 nm using a microplate reader (SynergyTM HTX Multi-Mode Reader, BioTek, Winooski, VT, USA). Different treatment durations were used based on previous studies and because MTT assay outcomes depend on cell growth characteristics, metabolic activity, and assay duration. In addition, the viability assay for each cell type was aligned with the time window used in the corresponding downstream experiments: B16F10 cells were assessed under the 72 h melanogenesis-related treatment condition, whereas HaCaT cells were assessed under the 24 h UVB-related photodamage condition.

### 4.7. Measurement of Intracellular ROS Level

Intracellular ROS level was measured using the DCFH-DA method with some modifications [65]. HaCaT cells were seeded in a 96-well plate at a density of 1.0 × 10^4^ cells/well and incubated overnight. After incubation and pretreatment with PAEE (100, 200, and 400 μg/mL), the control group and a UVB-only group were included. After 24 h of incubation, the cells were exposed to UVB irradiation (30 mJ/cm^2^), a dose selected with reference to a previous study, followed by treatment with DCF-DA solution (50 μM) for 30 min in the dark. The fluorescence intensity was then measured using a microplate reader (Synergy™ HTX Multi-Mode Reader, BioTek, Winooski, VT, USA) to evaluate ROS generation in HaCaT cells.

### 4.8. Measurement of Melanin Content

Melanin content was quantified using a slightly modified version of a previously reported method [66]. Briefly, B16F10 cells were seeded at a density of 2.5 × 10^4^ cells/well in 6-well plates and pretreated with α-MSH (100 nM) for 1 h. Cells were then treated with PAEE (200, 400, and 800 μg/mL) and arbutin (200 μg/mL); a control group and an α-MSH-only group were included. After 72h of incubation, cells were washed twice with PBS, followed by the addition of 200 μL of 1× trypsin and 800 μL of PBS. The cells were collected by centrifugation at 13,000× *g* for 10 min. The resulting pellet was dissolved in 1 N NaOH containing 10% DMSO and incubated at 80 °C for 1 h to solubilize melanin. Absorbance was measured at 475 nm using a microplate reader (Synergy™ HTX Multi-Mode Reader, BioTek, Winooski, VT, USA).

### 4.9. Measurement of Tyrosinase Activity

Tyrosinase activity was quantified using a slightly modified version of a previously reported method [67]. B16F10 cells were seeded at a density of 2.5 × 10^4^ cells/well in 6-well plates and pretreated with α-MSH (100 nM) for 1 h. Cells were then treated with PAEE (200, 400, and 800 μg/mL) and arbutin (200 μg/mL); the control group and an α-MSH-only group were included. After 72 h of incubation, cells were washed twice with PBS and lysed in 50 mM sodium phosphate buffer (pH 6.8). The cell lysates were collected by centrifugation at 12,000 rpm for 30 min at 4 °C. After protein quantification and standardization, 80 μL of the supernatant was transferred to 96-well plates and mixed with 10 mM L-DOPA. The reaction mixture was incubated at 37 °C for 1 h, and absorbance was measured at 492 nm using a microplate reader (Synergy™ HTX Multi-Mode Reader, BioTek, Winooski, VT, USA).

### 4.10. Enzyme-Linked Immunosorbent Assay (ELISA)

HaCaT cells were seeded in 6-well plates at a density of 1 × 10^5^ cells/well and incubated overnight. After incubation, the cells were irradiated with 30 mJ/cm^2^ UVB. After irradiation, cells were treated with PAEE (100, 200, and 400 μg/mL); the control group and a UVB-only group were included. After 24 h of incubation, culture supernatants were collected, and human IL-1β levels were measured using the Quantikine^®^ ELISA Kit (Human IL-1β/IL-1F2, Catalog #DLB50; R&D Systems, Minneapolis, MN, USA) according to the manufacturer’s instructions.

### 4.11. Western Blot

HaCaT cells were seeded in 6-well plates at a density of 1 × 10^5^ cells/well and exposed to UVB (30 mJ/cm^2^). Cells were then treated with PAEE (100, 200, and 400 μg/mL), and control and UVB-only groups were included for 24 h to evaluate the expression of filaggrin and involucrin, as well as MMP-1, a UV-responsive matrix-degrading enzyme associated with photodamage. B16F10 cells were seeded in 6-well plates at a density of 2.5 × 10^4^ cells/well and pretreated with α-MSH (100 nM) for 1 h. Cells were then treated with PAEE (200, 400, and 800 μg/mL), and control, α-MSH-only, and arbutin-treated groups were included for 72 h to evaluate the expression of melanogenesis-related proteins, including PKA, p-PKA, CREB, p-CREB, MITF, TRP-1, and tyrosinase, with GAPDH used as a loading control.

Both cells were washed with cold PBS and lysed using M-PER™ Mammalian Protein Extraction Reagent (Thermo Fisher Scientific, Waltham, MA, USA) and centrifuged to obtain cell lysates. Western blot analysis was performed in accordance with our previous study [68]. Equal amounts of protein (50 μg per lane) were separated by SDS-PAGE and transferred onto nitrocellulose (NC) membranes. The membranes were blocked with 5% skim milk in Tris-buffered saline containing Tween 20 (TBST) for 1 h at room temperature and incubated overnight at 4 °C with primary antibodies at the manufacturer-recommended dilutions. After washing, the membranes were incubated with HRP-conjugated secondary antibodies for 1 h at room temperature, and protein bands were visualized using enhanced chemiluminescence. Band intensities were quantified using ImageJ software (version 1.53e; National Institutes of Health, USA) For signaling proteins, both phosphorylated and total forms were analyzed to distinguish changes in phosphorylation status from changes in total protein abundance. Phosphorylated protein levels were normalized to their corresponding total proteins.

### 4.12. Maintenance of Parental Zebrafish

Zebrafish were purchased from Sin-yong Aquarium (Cheonan, Republic of Korea) and maintained in accordance with our previous study [69]. Zebrafish were maintained in 3 L acrylic tanks at 28.5 °C under a 14 h light/10 h dark cycle in system water (pH 7.0–7.5, conductivity 500–1500 µS/cm). Fish were fed three times daily. Embryos were obtained from breeding groups consisting of two males and two females, with spawning induced in the morning by light exposure. Embryos were collected within 30 min in Petri dishes.

### 4.13. Toxicology Evaluation in Zebrafish

Toxicology evaluation involved the assessment of survival rates and morphological changes. The survival rate and changes in pigmentation and morphology were examined at 3 days post-fertilization (dpf). At 3 dpf, zebrafish embryos were treated with PAEE (100, 200, and 400 μg/mL) and the control group was included; the embryos were anesthetized using a tricaine methanesulfonate solution (Sigma, St. Louis, MO, USA) and subsequently imaged under a stereomicroscope (Nikon SMZ745T; Nikon Instruments, Tokyo, Japan). Survival rate and morphological toxicity (body length, yolk edema size, and cardiac size) were measured in a representative subset of five embryos per group. Morphological parameters were quantified from stereomicroscopic images using ImageJ software (version 1.53e; National Institutes of Health, USA).

### 4.14. Melanin Contents of Zebrafish Embryos

Melanin content in zebrafish embryos was determined based on a previously reported method [70]. Briefly, 0 dpf zebrafish embryos were seeded in a 6-well plate and pretreated with α-MSH (100 nM) for 1 h. After pretreatment, the embryos were treated with PAEE (100, 200, and 400 μg/mL) and arbutin (200 μg/mL), and the control group and an α-MSH-only group were included for 72h. After treatment (3 dpf), zebrafish embryos at 3 dpf were sonicated using T-PER™ Tissue Protein Extraction Reagent (Thermo Fisher Scientific, Waltham, MA, USA). Following centrifugation, the resulting pellet was resuspended in 500 μL of 1 N NaOH and incubated at 100 °C for 30 min. The mixture was then vortexed to fully dissolve the melanin. The optical density of the supernatant was subsequently measured at 490 nm using a microplate reader (Synergy™ HTX Multi-Mode Reader, BioTek, Winooski, VT, USA).

### 4.15. Measurement of Zebrafish ROS Level

Zebrafish ROS levels were determined based on a previously reported method [71]. Briefly, 0 dpf zebrafish embryos were seeded in a 6-well plate and pre-treated with PAEE (100, 200, and 400 μg/mL), and the control group and a UVB-only group were included. Immediately, the embryos were exposed to UVB irradiation (30 mJ/cm^2^) at 0, 1, and 2 dpf. After 72 h of pretreatment (3 dpf), the embryos were washed and incubated with DCFH-DA solution (25 μg/mL) for 30 min in the dark. The fluorescence intensity was then measured using an EVOS fluorescence microscope (Invitrogen, Thermo Fisher Scientific, Waltham, MA, USA) to evaluate ROS generation in zebrafish. Fluorescence intensity was quantified from captured images and normalized to the control group using ImageJ software (version 1.53e; National Institutes of Health, USA).

### 4.16. Statistical Analysis

All experiments were performed in triplicate, and data are presented as the mean ± standard deviation (SD). Statistical analyses were performed using GraphPad Prism 8 (GraphPad Software, San Diego, CA, USA). One-way analysis of variance (ANOVA) followed by Dunnett’s multiple comparisons test was used. Adjusted *p* values were applied for multiple comparisons, and *p* < 0.05 was considered statistically significant.

## Figures and Tables

**Table 1:** Compounds in the LC-Q-TOF-MS/MS profile of PAEE.

NO.	Name	RT (min)	Formula	Mass Error (ppm)	Transition (m/z)
1	Prekinamycin	0.88	C_18_H_10_N_2_O_4_	−3.33124	317.0557
2	Lactitol	0.93	C_12_H_26_O_12_	−1.61695	343.124
3	2-(α-D-mannosyl)-D-glyceric acid	0.93	C_9_H_16_O_9_	0.507539	267.0723
4	Tolmetin sodium	3.47	C_15_H_18_NNaO_5_	−3.31231	296.0894
5	Tuliposide A	3.97	C_11_H_18_O_8_	−1.85922	277.0924
6	1,5-Pentanedisulfonyl difluoride	4.28	C_5_H_10_F_2_O_4_S_2_	−1.99302	216.9805
7	eucommiol	8.16	C_9_H_16_O_4_	−3.62659	187.0969
8	pinellic acid	11.31	C_18_H_34_O_5_	−0.49086	329.2332
9	Benzenamine, N,N (1,3-dimethyl-1-propen-1-yl-3- ylidene)bis[2,6-bis(1-methylethyl)-, aluminum salt (1:1)	17.36	C_29_H_41_AlN_2_	1.73116	487.2912
10	(E)-4,8-Dimesityl-6,6-dimethyl-2-styryl-1-oxa-4,8- diazaspiro[2.5]octane-5,7-dione	17.64	C_33_H_36_N_2_O_3_	−2.89526	553.2693
11	17β-Hydroxy-2α-(hydroxymethyl)-5α-androstan-3-one	17.84	C_20_H_32_O_3_	−2.61961	319.227
12	α-D-Glucopyranosyl 6-O-palmitoyl-α-D-glucopyranoside	18.34	C_28_H_52_O_12_	−0.78916	561.3276
13	N,N,N,N Tetrabenzyl-1,2-diphenylethane-1,2-diamine	18.36	C_42_H_40_N_2_	−0.0442	553.3013
14	Methoxy(dimethyl)[2-(4,4,5,5-tetramethyl-1,3,2- dioxaborolan-2-yl)-6-hepten-3-yl]silane	18.64	C_16_H_33_BO_3_Si	0.224731	293.2114
15	10,16-Dihydroxyhexadecanoic acid	18.93	C_16_H_32_O_4_	0.538826	287.2229

## Data Availability

The original contributions presented in this study are included in the article. Further inquiries can be directed to the corresponding authors.
